# A71 IMPACT OF THE COVID-19 PANDEMIC ON WAIT TIMES, AND CLINICALLY RELEVANT FINDINGS AT COLONOSCOPY

**DOI:** 10.1093/jcag/gwad061.071

**Published:** 2024-02-14

**Authors:** A Barkun, K Ravanbakhsh, D Kim, G Milky, P Stanowski, O Geraci, M Martel, C Menard, D von Renteln

**Affiliations:** McGill University Health Centre, Montreal, QC, Canada; Research Institute of the McGill University Health Centre, Montreal, QC, Canada; McGill University Health Centre, Montreal, QC, Canada; Research Institute of the McGill University Health Centre, Montreal, QC, Canada; Research Institute of the McGill University Health Centre, Montreal, QC, Canada; Research Institute of the McGill University Health Centre, Montreal, QC, Canada; Research Institute of the McGill University Health Centre, Montreal, QC, Canada; Universite de Sherbrooke, Sherbrooke, QC, Canada; Centre Hospitalier de l'Universite de Montreal, Montreal, QC, Canada

## Abstract

**Background:**

The widespread use of a standardized and validated province-wide colonoscopy referral form (PCRF), regrouping mutually exclusive indications into suggested priority wait times categories (P1 to P5), has allowed for a more comprehensive description of routine colonoscopy practice.

**Aims:**

To better understand the impact of COVID-19 on the routine practice of colonoscopy.

**Methods:**

This is a multicenter retrospective cohort study of consecutive adult patients referred with PCRF data available from two Quebec tertiary hospitals. Patient and procedural characteristics were recorded. The primary outcomes were the diagnostic rates of colorectal cancers (CRC) and clinically significant lesions (CSF), defined as endoscopic findings affecting subsequent patient management, excluding hemorrhoids and diverticulosis. The secondary outcome was procedural wait times. We compared endpoints contrasting colonoscopy findings pre-COVID (before March 15th, 2020) to intra-COVID (after April 15th, 2020).

**Results:**

7,476 pre-COVID and 7,181 Intra-COVID patients (mean age 59.2 ± 14.0 years, 50.9% female) were included from 2018 to 2022. There were no clinically relevant between-group differences in patient characteristics. CRC detection remained similar (0.9% pre- vs 0.8% intra-COVID, p=0.69), while CSF were diagnosed more frequently intra-COVID (41,2% vs 39.7%, p=0.02).

There were higher rates of indications performed for urgent and semi-elective priorities (P2, P3) intra-COVID (2.9% vs 1.3%, pampersand:003C0.01, and 50.5% vs 47.9%, pampersand:003C0.01). Corresponding intra- vs pre-COVID differences in indications (all Pampersand:003C0.01) included a clinical suspicion of active inflammatory bowel disease (6.4% vs 5.2%), a high index of suspicion for cancer based on imaging, endoscopy or clinical exam (2.9% vs 1.3%), suspicion of occult colorectal cancer (1.4% vs 0.9%), and doing a repeat endoscopy because of a prior inadequate bowel preparation (1.3% vs 0.8%). In contradistinction, more elective colonoscopies had been performed pre-COVID (P4: 15.5% vs 8.6%, pampersand:003C0.01, and P5: 5.2% vs 3.8%, pampersand:003C0.01. COVID Colonoscopy wait times grew significantly longer intra- vs pre-COVID (176.3 ± 252.4 days vs 78.6 ± 110.2 days, pampersand:003C0.01).

**Conclusions:**

We witnessed significant changes in indications and referral priorities distributions for colonoscopy pre- vs intra-COVID, amidst longer wait times. These practice modifications did not alter CRC diagnostic yields, but resulted in greater proportions of CSF detection

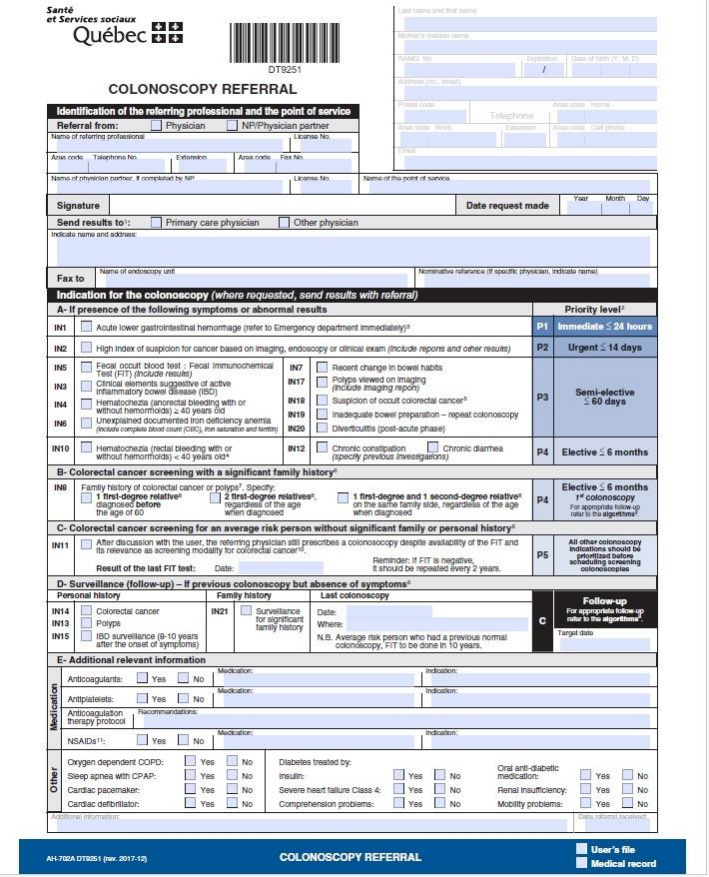

**Funding Agencies:**

CPAC and MSSS

